# Assessment of hepatoprotective, nephroprotective efficacy, and antioxidative potential of *Moringa oleifera* leaf powder and ethanolic extract against PCOS‐induced female albino mice (*Mus Musculus*)

**DOI:** 10.1002/fsn3.3646

**Published:** 2023-09-01

**Authors:** Shakeela Khalid, Muhammad Arshad, Komal Raza, Shahid Mahmood, Farzana Siddique, Nida Aziz, Sarfraz Khan, Waseem Khalid, Ammar AL‐Farga, Faisal Aqlan

**Affiliations:** ^1^ Department of Zoology University of Sargodha Sargodha Pakistan; ^2^ Liver Center District Headquarter Hospital Faisalabad Pakistan; ^3^ Institute of Food Science and Nutrition University of Sargodha Sargodha Pakistan; ^4^ Department of Chemistry Air Base College Sargodha Pakistan; ^5^ University Institute of Food Science and Technology The University of Lahore Lahore Pakistan; ^6^ Department of Biochemistry, College of Sciences University of Jeddah Jeddah Saudi Arabia; ^7^ Department of Chemistry, College of Sciences Ibb University Ibb Yemen

**Keywords:** LFT, MDA, metformin, *moringa oleifera*, RFT, testosterone enanthate

## Abstract

*Moringa oleifera* is a medicinal plant that has anti‐inflammatory, antihypertensive, antidiabetic, tissue‐protective, and antioxidant activities. Here, we evaluated the protective effect of *M. oleifera* leaf powder (MoLP) and 70% ethanol *M. oleifera* leaf extract (MoLE) on mitigating polycystic ovary syndrome (PCOS)‐induced liver and kidney dysfunction via regulating oxidative stress in female albino mice (*Mus musculus*). The efficacy of *M. oleifera* was compared with metformin (standard medicine used to treat infertility in women). PCOS was induced by intramuscular injection of testosterone enanthate at 1.0 mg/100 g BW for 35 days. PCOS‐induced mice were treated with MoLP (250 and 500 mg/Kg), MoLE (250 and 500 mg/kg), and metformin (250 mg/kg) orally for 14 days. Renal function test (RFT), liver function test (LFT), and oxidative stress biomarker malondialdehyde (MDA) were quantified in serum at 0, 7, and 14 days of intervention. Mice treated with *M. oleifera* and metformin showed a significant decrease (*p* < .001) in alanine aminotransferase (ALT), aspartate aminotransferase (AST), alkaline phosphate (ALP), total bilirubin, urea, creatinine, and a significant increase (*p* < .001) in total protein, albumin, globulin, and albumin/globulin (A/G) ratio. Oxidative stress decreased significantly (*p* = .00) with respect to treatments, exposure days, and their interaction in metformin and all *M. oleifera*‐treated groups. *M. oleifera* leaf powder and extract reduce oxidative stress and enhance nephron‐hepatic activity in PCOS‐induced female albino mice.

## Graphical abstract

This study highlights the potential of both *Moringa oleifera* leaf powder and extract as effective therapeutic options for managing PCOS, and liver and kidney diseases. It also provides new opportunities for further research in this field.

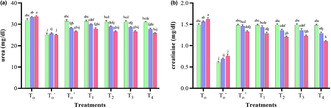

## 1 INTRODUCTION

Polycystic ovary syndrome (PCOS) is a complex disorder whose etiology seems to be polygenic, multifactorial, and multisystem hormonal problems. It affects approximately 5–10% of women during puberty and is characterized by elevated androgen levels, lack of ovulation, insulin resistance (IR), and type II diabetes (Broskey et al., 2018). Women with PCOS may have hyperinsulinemia, hyperlipidemia, and a high LH/FSH ratio that affects oocyte quality (Siahaan et al., 2022). The use of androgens (testosterone, testosterone propionate, enanthate) or estrogens (estradiol valerate) is a technique to develop a type of physiopathology in animal models similar to women with PCOS (Anesetti & Chávez‐Genaro, 2016). Insulin resistance (IR) due to elevated levels of free fatty acids and hyperglycemia leads to the activation of reactive oxygen species (ROS) and enhances oxidative stress (Velaga et al., 2017).

Oxidative stress is caused by a deskbound lifestyle, obesity, and exposure to toxic compounds, pesticides, and heavy metals, which raises the risk of diabetes, metabolic disorders, inflammation, and infertility (Kostoff et al., 2020; Tsatsakis et al., 2019). Oxygen free radicals are superoxides that can cause cell damage and apoptosis, leading to cancer and myocardial infarction (Padureanu et al., 2019; Tsatsakis et al., 2019). Oxidative stress also results in PCOS and leads to a high level of malondialdehyde (MDA) (Kalhori et al., 2018). The presence of toxic substances in the body, such as MDA, free radicals, and other lipid peroxidation products causes oxidative stress and can contribute to insulin resistance (IR) (Murri et al., 2013).

PCOS patients are increased risk of diabetes, abdominal adiposity, and metabolic disorders like liver and kidney damage and steatosis (Vassilatou, 2014). In women with PCOS, IR, and hyperandrogenism, double the chances of fatty liver disease (steatosis), which can cause more severe and rapidly progressive liver disease at a young age (Javed et al., 2019). PCOS can cause kidney cysts that cause enlarged kidneys and loss of function. However, women with PCOS may have an increased risk of developing chronic kidney disease (CKD). CKD damages renal activity, ultimately leading to end‐stage kidney function, and is one of the leading causes of mortality (Fraser et al., 2015). Several studies have shown that decreased glomerular filtration and microalbumin in urea are vital markers of kidney dysfunction in PCOS women (Song et al., 2019). Escobar‐Morreale (2018) found more cases of age‐dependent CKD in PCOS‐induced female rats.

Metformin is an antidiabetic drug that shows redox‐modulating effects and addresses numerous ailments linked with oxidative stress, and can chelate polyvalent metal ions, reduce oxidative stress, and mitigate toxicity (Karmanova et al., 2023). Metformin, clomiphene citrate, and tamoxifen are commonly prescribed for PCOS. Studies have shown that metformin ameliorates hormonal disorders, maintains ovarian physiology, and reduces obesity in PCOS patients by inhibiting hepatic glucose uptake and promoting peripheral glucose uptake (Chukwunonso Obi et al., 2016). Long‐term use of these drugs may have limited efficacy and cause many side effects, such as abdominal disturbance, nausea, headache, and weight gain (Domecq et al., 2013; Younas et al., 2022). However, it is necessary to identify and develop alternative and safe sources to prevent and treat PCOS. This is a leading cause of infertility and oxidative stress (Jelodar & Askari, 2012).

*Moringa oleifera* is a food plant and is often called golden tree, magic plant, drumstick, and sohanjna (Grosshagauer et al., 2021). It is grown for its edible leaves and flowers, nutritious pods, and extensively consumed as food, medicines, cosmetic oil, or fodder for livestock (Vergara‐Jimenez et al., 2017). It contains multiple therapeutically active chemicals that make it an ideal candidate for supplementation with side effects suppression. Historically, it has been used as an antidiabetic, antioxidant, antimicrobial, and anti‐inflammatory remedy to cure more than 300 diseases (Zucca et al., 2016). It is a storehouse of nutrients, such as minerals, vitamins, flavonoids, and phenols. Flavonoids like quercetin possess strong antioxidant properties, inhibit various enzymes, quench ROS production, and chelate all the metals used in radical chain reactions (Banafsheh & Sirous, 2016; Kumar & Pandey, 2013; Shah & Patel, 2016). Polyphenols (gallic acid) have antiobesity, antidiabetic, anti‐inflammatory, and antioxidative activity (Esmaeilzadeh et al., 2020). Chlorogenic acid (CGA) elevates nonenzymatic antioxidant activity. Ferulic acid is a strong antioxidant that provides lipid peroxidation protection, scavengers free radicals, and binds to Fe and Cu (Tee‐ngam et al., 2013; Zduńska et al., 2018). Sinapic acid and p‐Coumaric acid possess anti‐inflammatory, antimicrobial, antimutagenic, antioxidant, antidiabetic, and antihyperlipidemic properties (Chen, 2016).

A kidney function test (RFT), such as urea and creatinine, and a liver function test (LFT) are biochemical blood tests designed to assess the working of the kidneys and liver. Examining hepatic injury in patients often involves analyzing the levels of alanine aminotransferase (ALT) and aspartate aminotransferase (AST), which are both important biomarkers. Albumin and transferase tests are related to cellular integrity, while alkaline phosphatase is linked to the biliary tract (McClatchey, 2002). A high level of BUN (blood urea nitrogen) is caused primarily by a high‐protein diet, congestive heart failure, gastrointestinal hemorrhage, and increased catabolism (Braunwald et al., 2005).

Considering the alarming prevalence of PCOS and poor physical, mental, and social outcomes, a study was carried out to investigate the hepatoprotective and nephroprotective efficacy of *M. oleifera* leaf powder (MoLP) and *M. oleifera* leaf extract (MoLE) against PCOS‐induced albino mice. We hypothesized that MoLP and MoLE are effective therapeutic interventions to reverse PCOS. We further presume that the effects of MoLP and MoLE will be comparable to metformin. Thus, it is possible that this could reverse the kidney and liver damage caused by PCOS in humans.

## 2 MATERIALS AND METHODS

### 2.1 Plant collection and preparation of aqueous and ethanolic extracts

*Moringa oleifera* fresh leaves were collected from the garden of the University of Sargodha. The detailed procedure for preparing aqueous and ethanol extract was previously described in Khalid et al. (2023).

To prepare the ethanol extract (MoLE), 300 g of the powder was macerated in 70% ethanol (5000 mL) using a shaking incubator. The resulting mixture was filtered, evaporated using a rotary evaporator under reduced pressure, and stored at 4°C. Seventy‐percent ethanolic extract of *M. oleifera* leaves (MoLE) was used as treatment dosage.

### 2.2 Development of basal and focused feed

The feed was prepared following proximate and HPLC analyses of MOLP extracts. Basal feed was composed of carbohydrates (16%), proteins (18%), and fats (2%), while focused feed was prepared as per table.

### 2.3 Experimental animals

For the experiment, 100 female albino mice (*Mus musculus*) were used. They were 8–9 weeks old and weighed 26 ± 2 g. Mice were obtained from the stock of mice bred in the animal house of the University of Sargodha, Pakistan. They were kept under standard housing conditions with a temperature of 22 ± 2°C, relative humidity of 45 ± 5%, and 12 h of light/dark cycles. Before the experiment, the mice were acclimatized for 10 days. Mice with anatomical abnormalities or pregnancies were excluded from the experiment during the acclimatization days (Amelia et al., 2018). Ten albino mice were randomly selected, and their biomarker, including malondialdehyde (MDA), renal function test (RFT), and liver function test (LFT), were measured.

### 2.4 Induction of PCOS

After a period of acclimatization, 1.0 mg/100 g BW testosterone enanthate was dissolved in sesame oil (100:9900 μL), and injected in into thigh muscle of albino mice for 35 days to induce PCOS (Kalhori et al., 2018).

### 2.5 Experimental design

PCOS‐induced albino mice (*n* = 76) were randomly divided into six treatment groups, while non‐PCOS‐induced mice (*n* = 10) were kept as a negative control group. The negative control group (non‐PCOS‐induced mice) was assigned to group $T_{0}^{-}$. PCOS‐induced mice were randomly divided into six groups *T*_0_ (Placebo), $T_{0}^{+}$ (Positive control, Basal Feed + Metformin at 250 mg/kg BW), *T*_1_ (MoLP‐250, Basal Feed + MoLP at 250 mg/kg BW), *T*_2_ (MoLP‐500, Basal Feed + MoLP at 500 mg/kg BW), *T*_3_ (MoLE‐250, Basal Feed +70% MoLE at 250 mg/kg BW), and *T*_4_ (MoLE‐500, Basal Feed +70% MoLE at 500 mg/kg BW). The study comprised 10 days of acclimatization, 35 days of induction, and 14 days of intervention (Amelia et al., 2018).

### 2.6 Biochemical analysis

Various biomarkers of albino mice were studied during acclimatization, PCOS induction, intervention period, and at the completion of the study.

### 2.7 Interventions among infertile albino mice

The biomarkers were reexamined at the end of the experiment in all treatment groups by following treatment for 14 days in PCOS‐induced albino mice.

### 2.8 Lipid peroxidation

Malondialdehyde (MDA) concentration was determined using the Buege and Aust (1978) methodology based on thiobarbituric acid reactivity. A volume of 50 μL of serum was taken, and 1 mL of trichloroacetic acid TCA‐2‐thiobarbituric acid (TBA)–HCl reagent was added, and then the samples were placed in boiling water for 10 min. After cooling, the reaction mixture was centrifuged, and the supernatant was separated. The absorbance was read at 535 nm. 1.56 × 105/mol/cm as the molar absorbance coefficient was used to calculate MDA concentration (Khalofah et al., 2020).

### 2.9 Safety biomarkers

To ensure the safety of interventions on albino mice, blood samples were collected to investigate LFT and RFT at the University Medical and Diagnostic Center (UMDC) at 0, 7, and 14 days.

#### 2.9.1 Liver function test (LFT)

Alanine aminotransferase (ALT) and aspartate aminotransferase (AST) were determined using the method of Reitman and Frankel (1957). ALT and AST were analyzed by monitoring the concentrations of pyruvate hydrazine and oxaloacetate hydrazine, respectively, using 2,4 dinitrophenylhydrazine. For the experiment, 0.5 mL of ALT and AST substrate phosphate buffer solutions was mixed with 0.1 mL of serum in test tubes. In blank test tubes, 0.1 mL of distilled water was poured instead of serum. These test tubes were incubated in a water bath at 37°C for 25 min. Then, 2,4 dinitrophenylhydrazine (0.5 mL) was added to all test tubes, and the blank test tubes received only a 0.1 mL of sample, which was allowed to stand at room temperature for 20 min. In the end, 5.0 mL of NaOH was pipetted into both test tubes. The absorbance of the sample was recorded at 550 nm after 5 min.

Alkaline phosphate (ALP) was detected by ELITech kits as described by Young (1997). ALP concentration was measured using 2‐amino, 2‐methyl, 1‐propanol (AMP), and p‐nitrophenyl phosphate as substrates. A volume of 200 μL of ALP substrate phosphate buffer solutions and 5 μL serum were added to sample test tubes. In blank test tubes, 200‐μL AMP substrate phosphate buffer solutions and 5‐μL distilled water were poured using a pipette. Both test tubes were incubated in a water bath at 37°C for 15 min. After incubation, the sample absorbance was recorded at 405 nm after 5 min.

Total protein, albumin, globulin, and albumin/globulin (A/G) ratio were evaluated using the method of Lowry et al. (1951). Protein was assessed by arranging different sets of test tubes in racks and filled with bovine serum albumin (BSA) with concentrations of 0, 10, 20, 30, 40, 50, 60, 70, 80, 90, and 100 μL. Two milliliters of solution (48 mL of 2% Na_2_CO_3_ in 0.1 mL NaOH; 1 mL of 0.5% CuSO_4_. 5H_2_O in water) and 1 mL of 1% NAK Tartarate in water were incubated at 25°C for 10 min. In the end, 0.2 mL of diluted Folin phenol was mixed into each test tube, vortexed, incubated for 25 min, and the absorbance was recorded at 600 nm.

Total bilirubin was measured using Jendrasskik and Grof (1938). A volume of 0.2 mL of sulfanilic acid was poured into the sample test tube and the blank test tube by a pipette. A volume of 0.05 mL of sodium nitrite was added to the sample tube. Ten milliliters of caffeine and 0.2 mL of sample were added to both test tubes. They were mixed thoroughly and incubated at 25°C for 10 min. Tartrate (1.0 mL) was poured in the end and incubated at 25°C for 25 min, and the absorbance of the sample was measured at 578 nm (Nwankwo et al., 2015).

#### 2.9.2 Renal function test (RFT)

Serum urea and creatinine were assessed using method of Tietz (1994). Serum urea was analyzed by taking 10 μL of sample in a sample test tube, 10‐μL standard solution in the standard test tube, and 10‐μL distilled water in the blank test tube. Ten‐microliter nitroprusside and urease were added to all three test tubes. Test tubes were vortexed and incubated at 37°C for 10 min. In the end, 2.0 mL of phenol was added to the test tubes, followed by 2.50 mL of sodium hypochlorite. The tubes were incubated at 37°C for 10 min. The sample absorbance was recorded at 546 nm (Nwankwo et al., 2015).

To measure creatinine concentration, 100 μL of the sample was added to the sample test tube, 100 μL of distilled water into the blank test tube, and 100 μL of the standard solution in the standard test tube. The tubes were vortexed after adding standard reagent, and the absorbance of the sample was measured at 492 nm (Nwankwo et al., 2015).

### 2.10 Data analysis and statistical application

All results were expressed as mean ± SEM of ten replicates. The experimental data were tested for normal distribution with Kolmogorov's test. Multifactor analysis of variance (two‐way ANOVA) with ‘treatments’ and ‘days’ as independent variables was applied to test the main effects and interactions (Benjamini & Braun, 2002; Jaccard et al., 1984; Sawyer, 2009).

## 3 RESULTS AND DISCUSSION

### 3.1 Lipid peroxidation (nM/mL)

The oxidative stress in the current study is measured by an increase in malondialdehyde (MDA), a marker of lipid peroxidation. Serum MDA concentration was significantly increased with time in PCOS‐induced (*T*_0_) albino mice compared to the negative control group ($T_{0}^{-}$), as shown in Table 1. Increased testosterone levels caused oxidative stress in albino mice. Rojas et al. (2014) observed that oxidative stress causes infertility and heterogeneous disorders in PCOS patients. Murri et al. (2013) further elaborated that under oxidative stress, the production of highly poisonous products such as MDA, free radicals, and other lipid peroxidation products results in insulin resistance.

**TABLE 1:** Mean values indicating the effect of treatments and days on MDA (nM/mL) of albino mice.

Days				
Treatments	0	7	14	Means ± SEM
*T*_0_	869.5 ± 11.54^c^	1142.6 ± 15.17^b^	1213.8 ± 26.46^a^	1075.3 ± 104.93^A^
*T*_0_^−^	258.69 ± 3.84^h^	261.09 ± 4.85^h^	264.1 ± 5.85^h^	261.29 ± 1.56^F^
$T_{0}^{+}$	871.08 ± 8.96^c^	785.72 ± 4.09^d^	504.01 ± 6.01^f^	720.27 ± 110.90^B^
*T*_1_	868.39 ± 5.12^c^	681.8 ± 5.53^e^	493.09 ± 4^f^	681.09 ± 108.34^C^
*T*_2_	870.25 ± 4.53^c^	489.78 ± 3.67^f^	391.83 ± 4.6^g^	583.95 ± 145.91^D^
*T*_3_	878.44 ± 9.23^c^	479.99 ± 3.22^f^	383.1 ± 3.86^g^	580.51 ± 151.56^D^
*T*_4_	871.33 ± 4.65^c^	383.09 ± 3.8^g^	298.51 ± 4.73^h^	517.64 ± 178.52^E^
Means ± SEM	783.95 ± 87.55^A^	603.44 ± 111.69^B^	506.92 ± 122.56^C^	

*Note*: *T*_0_ = Basal Feed +5% Tween 80; $T_{0}^{-}$ = Basal Feed; $T_{0}^{+}$ = Basal Feed + Metformin at 250 mg/kg BW; *T*_1_ = Basal Feed + MoLP at 250 mg/kg BW; *T*_2_ = Basal Feed + MoLP at 500 mg/kg BW; *T*_3_ = Basal Feed + MoLE at 250 mg/kg BW; *T*_4_ = Basal Feed + MoLE at 500 mg/kg BW.

Oxidative stress decreased significantly (*p* = .00) with respect to treatments, exposure days, and their interaction in the metformin group ($T_{0}^{+}$) and all *M. oleifera*‐treated groups from *T*_1_ to *T*_4_ (Table S1). The lowest MDA level was recorded during *T*_4_ treatment (298.51 ± 4.73 nM/mL), where it was almost four times lower than *T*_0_ (1213.8 ± 26.46 nM/mL) after 14 days of exposure. Research by Siahaan et al. (2022) proved that *M. oleifera* leaves reduced IR and diabetes. Moreover, moringa extract is a powerful antioxidant that binds to reactive oxygen species (ROS) and limits oxidative stress in PCOS‐induced rats. Previous studies suggested that antioxidant treatment improves insulin function in diabetic patients by reducing oxidative stress. *T*_0_ subjects treated with *T*_2_ and *T*_4_ at a dose of 500 mg/kg BW showed significantly less oxidative stress, as shown in Table 1. Singh et al. (2009) claimed that flavonoids and phenols in plant leaves were antioxidants and inhibited lipid peroxidation.

In the present research, the MDA level of $T_{0}^{+}$ (504.01 ± 6.01 nM/mL) was approximately four times lower than *T*_0_ (1213.8 ± 26.46 nM/mL) on the 14th day of exposure (Table 1). Chukwunonso Obi et al. (2016) found that oxidative stress and free radicals cause diabetic complications, whereas metformin as an antioxidant helps combat these pitfalls. Several studies have shown that Moringa and metformin have a parallel relationship. However, Huang et al. (2015) reported that metformin long‐term administration causes indigestion in patients aggravating diarrhea and abdominal distress. With minimal side effects, herbal plants could be the best option. Gopalakrishnan et al. (2016) suggested that *M. oleifera* plant extract has the potential as antimicrobial, antioxidant, and antidiabetic.

### 3.2 Biosafety markers

Blood samples of mice from all treatment groups were collected and liver function test (LFT) and renal function test (RFT) were conducted at 0, 7, and 14 days.

#### 3.2.1 Liver function test (LFT)

The end product of lipid peroxidation, malondialdehyde (MDA), is concentrated in the liver of PCOS‐induced albino mice and enhances oxidative damage to lipids. MDA increased oxidative stress and impaired liver enzyme function compared to mice treated with metformin, *M. oleifera* leaf powder, and extract. Patients are diagnosed with liver disease by passing through a liver function test (LFT). Sustained liver injury causes hepatocellular injury and excessive collagen damage through the activation of hepatic satellite cells and liver fibrogenesis (Ahmed et al., 2018). A liver function test (LFT) is a biochemical blood assay designed to describe the working of the liver in the patient's body (Lee, 2009). Targher et al. (2016) stated that nonalcoholic fatty liver disease (NAFLD) and polycystic ovarian syndrome (PCOS) are related to each other. Oxidative stress and insulin resistance are common pathogenetic mechanisms in both diseases.

It has been shown in our study that antioxidants can reduce liver stress and PCOS. Metformin, *M. oleifera* leaf powder (MoLP), and *M. oleifera* leaf extract (MoLE) are used to establish the fact that they serve as protective antioxidant and antidiabetic drugs, thereby shielding the vital organs of the human body, such as the pancreas, liver, heart, and kidney from oxidative stress induced during PCOS complications. According to Altaee and Fadheel (2021), *M. oleifera* ethanol extracts inhibited lipid peroxidation and ROS in rabbits injected with iodide. Akter et al. (2021) found that *M. oleifera* extract limits free radical overproduction in rats induced by tilmicosin and Hg by inhibiting MDA concentration. ANOVA tables of different LFT parameters showed highly significant (*p* = .00) effects of treatments, days, and interaction (Tables S3–S10).

##### Serum aspartate transaminase (U/L)

**FIGURE 1:**
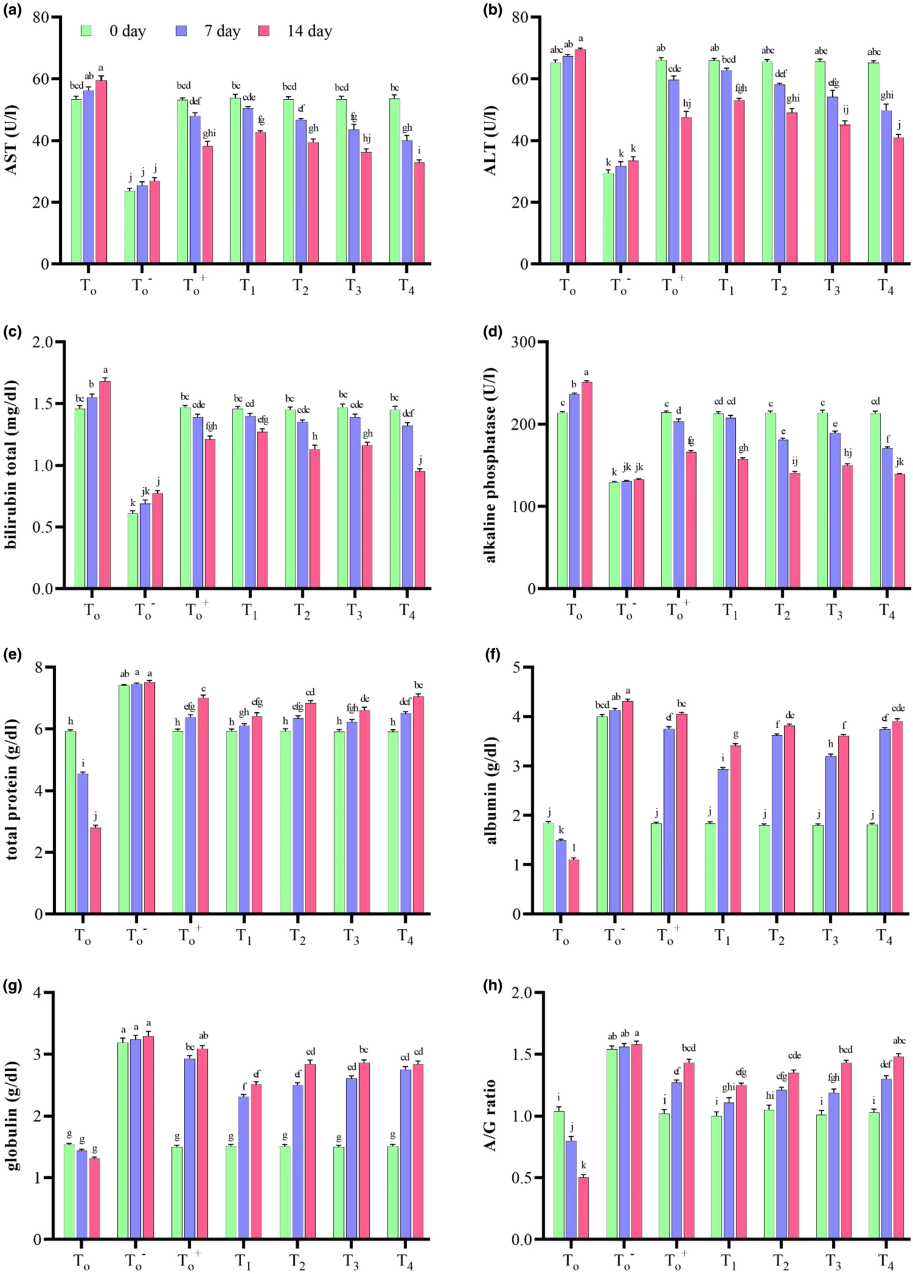
Effect of treatments and days on serum AST (a), ALT (b), bilirubin total (c), ALP (d), total protein (e), serum albumin (f), serum globulin (g), and A/G ratio (h) of albino mice (*n* = 10 mice per week). *T*_0_ = Basal Feed +5% Tween 80; $T_{0}^{-}$ = Basal Feed; $T_{0}^{+}$ = Basal Feed + Metformin at 250 mg/kg BW; *T*_1_ = Basal Feed + MoLP at 250 mg/kg BW; *T*_2_ = Basal Feed + MoLP at 500 mg/kg BW; *T*_3_ = Basal Feed + MoLE at 250 mg/kg BW; *T*_4_ = Basal Feed + MoLE at 500 mg/kg BW.

In albino mice, higher serum aspartate transaminase (AST) is associated with liver disease. A significant (*p* = .00) change in AST was observed due to treatments, days, and interactions (Table S2). There was an increase in AST levels in PCOS‐induced mice (*T*_0_) due to stress‐induced liver damage caused by injecting testosterone enanthate, with the highest value observed at day 14 (59.49 ± 1.49 U/L). Mice treated with *M. oleifera* and metformin showed a significant decrease in AST with time, which might be due to stress reduction. The lowest AST (32.89 ± 0.87 U/L) was found in *T*_4_ (MoLE extract 500 mg/kg BW) mice, as depicted in Figure 1a.

Vassilatou (2014) examined the high AST level in PCOS patients with fatty liver disease. According to another study (Schwimmer et al., 2005), elevated AST was found in 12% of 70 patients examined in an infertility clinic. Del Rio et al. (2005) claimed that serum catalase activity in diabetic rats was significantly lower than in rats treated with metformin. Iweala and Okeke (2005) also supported metformin's role in reducing oxidative stress induced by damaged liver physiology.

##### Serum alanine transaminase (U/L)

Higher serum ALT indicates liver disease in albino mice. A highly significant (*p* = .00) change in serum ALT in albino mice was observed due to treatments, days, and interactions (Table S3). Serum ALT increased significantly with time in PCOS‐induced mice (*T*_0_). The highest rise in serum ALT (69.5 ± 0.45 U/L) was seen in *T*_0_ mice may be due to stress imposed on the liver by injecting testosterone enanthate, and the lowest (40.9 ± 1.09 U/L) in *T*_4_ (MoLE extract 500 mg/kg BW) mice on day 14 might be due to stress reduction (Figure 1b). Macfarlane et al. (2000) explored that the rise in serum ALT is reflected in situations involving the necrosis of erythrocytes, myocardial cells, hepatocytes, and skeletal muscles.

Schwimmer et al. (2005) examined 70 female patients with PCOS in an infertility clinic and noted that 30% of females had high ALT levels in their serum. He found that most PCOS and liver disease females are insulin resistant and have abnormal ALT levels. These females with increased ALT had significantly (*p* < .01) more waist circumference, body mass index (BMI), total cholesterol‐to‐HDL‐cholesterol ratio, serum triglycerides, and degree of IR. Erbey et al. (2000) conducted a population‐based study including 18,825 adult women. The study recorded abnormal ALT in 2.2% of all women, 5.3% of diabetics, and 7.1% of obese women. Another research performed by Ruhl and Everhart (2003) on 5724 female patients showed that ALT increased with BMI: 1.0% normal body weight, 3.3% overweight, and 6.6% more in obese women.

##### Serum bilirubin total (mg/dL)

Higher serum levels of bilirubin indicate liver damage in albino mice. Highly significant (*p* = .00) changes in bilirubin in albino mice were observed due to treatments, days, and interaction (Table S4). High bilirubin levels were recorded in *T*_0_ over time. Statistical analysis revealed a highly significant (*p* = .00) decrease in serum bilirubin with time in albino mice treated with metformin and *M. oleifera* compared to mice with PCOS. The elevation in bilirubin (1.68 ± 0.29 mg/dL) was seen in *T*_0_ albino mice, which might be due to oxidative stress and reduction (0.95 ± 0.02 mg/dL) in *T*_4_ mice on day 14 of treatment is due to polyphenols in MoLE (Figure 1c).

Serum bilirubin is used as a diagnostic test for hepatic disorders. Its level depicts hepatotoxicity in humans (Saravanan et al., 2006). Serum bilirubin of more than 17 μmol/L indicates liver infection, and a level above 24 μmol/L shows abnormal liver laboratory tests (Thapa & Walia, 2007; Wong et al., 2004). Adewusi and Afolayan (2010) found that bilirubin levels were elevated in diseased groups due to hepatic damage and reduced in treatment groups by decreasing liver marker enzyme activity. The decrease in serum bilirubin after treatment with *Pelargonium reniforme* proved the efficacy of the plant‐based drug in regulating normal liver health. Abd‐Elhakim et al. (2021) emphasized in their study of rats that ethanol extract from *M. oleifera* detoxified plasma by lowering bilirubin.

Thus, plant extracts are enriched with antioxidants and free radical scavenging phenols, which have a strong reducing power. The results clearly showed that the plant was curative to alcohol‐induced liver damage by restoring bilirubin, liver marker enzymes, and other LFT parameters.

##### Serum alkaline phosphatase (U/L)

In albino mice, higher serum ALP causes liver damage. The change in serum ALP in albino mice was highly significant (*p* = .00) concerning treatments, days, and interaction (Table S5). The highest serum ALP (251.2 ± 1.68 U/L) was recorded in *T*_0_ mice after 14 days of exposure. PCOS‐induced mice treated with metformin and *M. oleifera* demonstrated a significant decrease in ALP with time. The lowest value (139.2 ± 0.72 U/L) was measured in the *T*_4_ group on the 14th day (Figure 1d).

According to Ojan and Nihorimbere (2004), an increase in serum ALP levels may be a sign of hepatobiliary diseases. Singha et al. (2007) have identified ALP as a sensitive marker enzyme for hepatocellular and liver damage. The serum of alcohol‐induced rats contained high levels of ALP, indicating aggravated permeability, damage, and necrosis. Furthermore, moringa promotes hepato‐protective effects against hepatocellular injury by attenuating serum ALP activities.

##### Serum total protein (g/dL)

Total protein levels varied significantly (*p* = .00) in all experimental groups with reference to treatments, days, and interactions (Table S6). Albino mice with PCOS showed a significant reduction in total protein with time (*T*_0_). The protein level of metformin ($T_{0}^{+}$) and all *M. oleifera*‐treated groups (*T*_1_–*T*_4_) significantly increased with time. The highest total protein (7.05 ± 0.08 g/dL) was recorded in *T*_4_ mice, which may be that MoLE reduced liver inflammation and oxidative stress, while the lowest level of total protein (2.8 ± 0.08 g/dL) was measured in *T*_0_ mice, as indicated by Figure 1e.

In a study carried out by Adewusi and Afolayan (2010), there was a significant decline in serum total protein levels in ethanol‐fed rats compared to the control group. The decline in total protein indicated a high level of cellular abnormality in chronic liver infection, and the rise in total protein justified liver inflammation recovery. This improvement is due to the phenols present in plant extract having strong antioxidative activity and redox properties. These phenols absorb and neutralize free radicals, quench atomic and triatomic oxygen, and decompose peroxides.

##### Serum albumin (g/dL)

Highly significant (*p* = .00) changes in albumin in albino mice were observed with treatments, days, and interactions (Table S7). PCOS‐induced mice showed decreased albumin levels with time, while mice treated with metformin and *M. oleifera* showed significant increases with time. The highest albumin (3.91 ± 0.05 g/dL) was measured in *T*_4_ mice due to the presence of antioxidants, gallic acid, sinapic acid, ferulic acid, and chlorogenic acid in MoLE and the least (1.1 ± 0.04 g/dL) in *T*_0_ group at 14th day of exposure (Figure 1f).

Albumin is a serum protein considered a biomarker of the nutrition and inflammation of the body (Don & Kaysen, 2004). The rise in albumin showed liver inflammation reduction. Cadet et al. (2012) claimed that chronic diseases and oxidative stress can be managed by selecting efficient antioxidants. Antioxidants are effective in countering oxidative stress and can inhibit, manage, and treat various pathologies (Pizzino et al., 2017). Antioxidants stop radical chain reactions, mitigating oxidative stress‐related damage.

##### Serum globulin (g/dL)

Highly significant (*p* = .00) differences in serum globulin in albino mice were recorded due to treatments, days, and interactions (Table S8). Albino mice with PCOS showed a decrease in globulin with time, while mice treated with metformin and *M. oleifera* showed a significant increase with time. The highest globulin (2.84 ± 0.05 g/dL) was measured in *T*_4_ and the least (1.31 ± 0.02 g/dL) in the *T*_0_ group on the 14th day of exposure (Figure 1g). Schiødt (2008) discussed globulin's multifunctional nature, as a patient with chronic liver disease has a low globulin level. Its function is to scavenge free radicals and control oxidative stress in hepatic tissues. Moringa extract may reduce liver inflammation and oxidative stress due to polyphenols and flavonoids. Moringa has a plethora of health benefits and improves the liver by raising globulin level.

Globulin is a reliable biomarker, a major protein in serum that is synthesized and secreted by plasma and hepatic cells in response to infection and inflammation. Inflammatory cytokines and antibodies are its two major constituents. Meyer et al. (2016) reported that patients with infection had high levels of globulin in their serum, indicating its role in immunity.

##### Serum albumin/globulin ratio

A highly significant (*p* = .00) variation in albumin‐to‐globulin ratio (A/G) in albino mice was studied due to treatments, days, and interaction (Table S9). Albino mice with PCOS showed a decrease in serum A/G ratio with time. In contrast, mice treated with metformin and *M. oleifera* showed a significant increase with time. The highest A/G ratio (1.48 ± 0.02) was recorded in the *T*_4_ mice, while the least (1.31 ± 0.02) in *T*_0_ mice, possibly due to disease severity, after 14 days of exposure (Figure 1h). The rise in globulin and A/G ratio indicated liver function recovery.

Hill et al. (2016) also demonstrated that the albumin‐to‐globulin ratio in patients decreases with infection and malignancy, suggesting their vital role in inflammation and the immune system. According to Yang et al. (2022), A/G is indicative of inflammation in the body, as well as stroke, infection, cancer, and autoimmune disorders. Yoshino et al. (2019) revealed that the opposite correlation between albumin and globulin decreases the A/G ratio. The A/G ratio is a measure of infection and inflammation, which indicates a higher level of infection.

#### 3.2.2 Renal function test (RFT)

The adverse symptoms of PCOS are hyperandrogenism, hypertension, insulin resistance, diabetes type 2, adiposity obesity, and hyperlipidemia, which results in infertility in women and irreversibly impairs renal physiology. Hyperandrogenism is correlated with kidney dysfunction and causes severe tubular epithelial cell injury in women with PCOS (Castro & Coresh, 2009; Webster et al., 2017). High testosterone in serum activates the apoptotic pathway in human renal tubule cells and increases kidney inflammation (Verzola et al., 2004). Sirmans and Pate (2014) found that insulin resistance in PCOS also leads to chronic kidney damage. Patil et al. (2017) observed renal function in hyperandrogenic female rats and reported a higher risk of kidney injury.

Kidney or renal function tests (RFT), such as urea and creatinine, are common laboratory tests used to study kidney function. Urea is a waste metabolite of protein catabolism by the liver. Allen (2012) describes creatinine as a crucial biosafety marker in renal health. It is produced during muscle catabolism and removed unchanged from the kidney. Song et al. (2019) investigated the link between kidney damage and PCOS. They selected 69 healthy and 55 PCOS women and assessed their testosterone levels and renal activity. Results showed that the urinary albumin‐to‐creatinine ratio in PCOS women was significantly higher than in non‐PCOS women, leading to kidney damage. They concluded that serum testosterone and PCOS cause kidney inflammation.

The present study demonstrated that *M. oleifera* is a preferred herbal remedy for treating kidney disorders, which is consistent with the findings of Saleh (2019). Akpan et al. (2018) proved that *M. oleifera* alleviated diabetic nephropathy in alloxan‐treated rats. Kagbo and Abaekwume (2021) reported that moringa taken at 500 mg/kg cured hepato‐renal toxicity caused by acetaminophen‐induced diabetes in *Rattus norvegicus*. Arafat et al. (2018) supported that *M. oleifera* leaf extracts decreased MDA, liver and kidney damage. Kakalij et al. (2014) documented that serum urea and serum creatinine can be used to measure drug‐induced nephrotoxicity. They claimed that nephrotoxicity in kidney activity is due to increased levels of blood urea, serum urea, serum creatinine, and urine creatinine.

Analysis of the variance of serum urea and creatinine was measured. Results indicated a highly significant (*p* = .00) reduction in urea and creatinine (Tables S10 and S11).

##### Serum urea (mg/dL)

**FIGURE 2:**
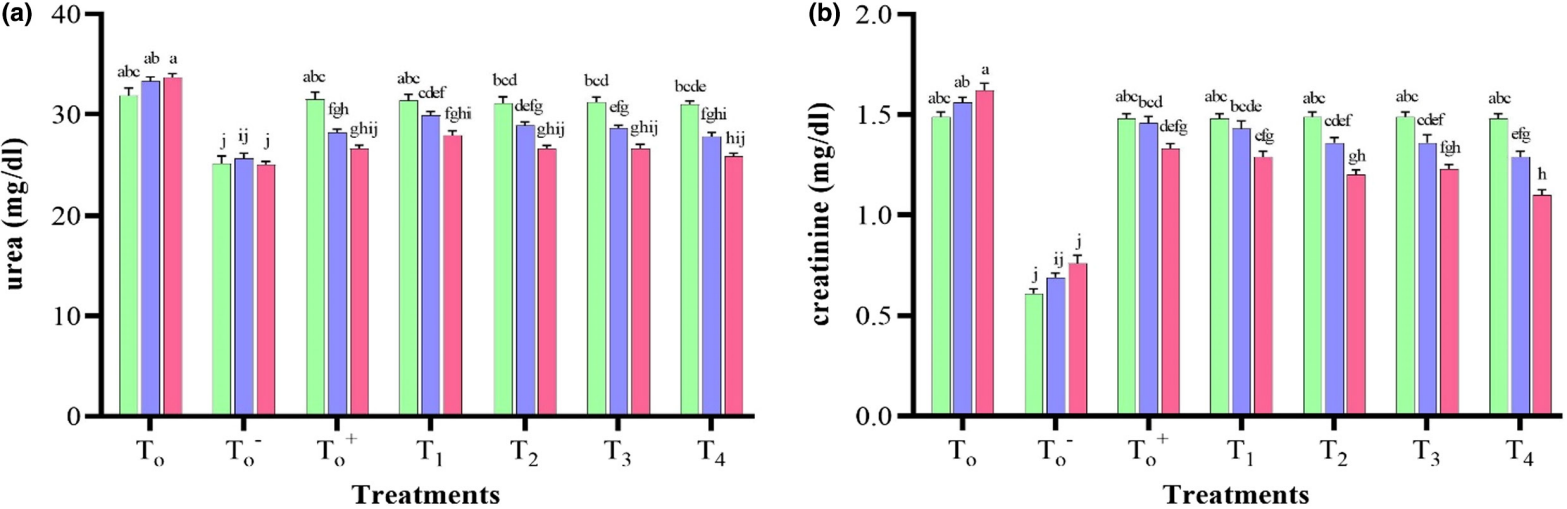
Effect of treatments and days on urea (a) and serum creatinine (b) of albino mice (*n* = 10 mice per week). *T*_0_ = Basal Feed +5% Tween 80; $T_{0}^{-}$ = Basal Feed; $T_{0}^{+}$ = Basal Feed + Metformin at 250 mg/kg BW; *T*_1_ = Basal Feed + MoLP at 250 mg/kg BW; *T*_2_ = Basal Feed + MoLP at 500 mg/kg BW; *T*_3_ = Basal Feed + MoLE at 250 mg/kg BW; *T*_4_ = Basal Feed + MoLE at 500 mg/kg BW.

A highly significant (*p* = .00) decline in serum urea in albino mice due to treatments, days, and their interaction was observed (Table S10). Serum urea concentration was significantly higher in *T*_0_ albino mice than $T_{0}^{-}$ with time. The highest serum urea (58.6 ± 1.09 mg/dL) was recorded in *T*_0_ mice due to PCOS induction and oxidative stress, and the least (25.8 ± 0.32 mg/dL) in *T*_4_ mice at the 14th day of exposure, indicating that *M. oleifera* leaf extract effectively restored the antioxidant activity of testosterone affected kidneys as presented in Figure 2a. There was a decline in urea, which indicated better renal function.

Previous studies have found that serum urea rises in response to glomerular injury and also in response to a decrease in glomerular filtration injuries (Manikandan et al., 2011). Ahmadvand et al. (2016) demonstrated that urea was four times higher in the serum of untreated nephrotoxic rats than in healthy rats. In their study, serum urea levels were significantly reduced in carvacrol treatment groups. As an antioxidant, carvacrol reduces kidney inflammation. Similarly, Akter et al. (2021) observed that oxidative stress and inflammation associated with kidney disease can be relieved by *M. oleifera*, an antioxidant food plant. They further suggested that moringa‐based medication would be a protective choice against various risk factors linked to renal pathologies.

##### Creatinine (mg/dL)

Highly significant (*p* = .00) variations in creatinine in albino mice were recorded due to treatments, days, and their interactions (Table S11). *M. oleifera* and metformin significantly decreased creatinine levels in PCOS‐induced mice. The highest creatinine level (1.62 ± 0.04 mg/dL) was recorded in *T*_0_ and the least (1.1 ± 0.02 mg/dL) in *T*_4_ on the 14th day of exposure (Figure 2b). The rise in creatinine showed poor kidney function may be due to decreased tubular secretion capacity, as supported by Traynor et al. (2006).

In the present study, MoLP and MoLE protected kidneys from PCOS‐induced testosterone imbalance by activating endogenous antioxidant systems and enzymatic mechanisms that counteract oxidative stress. Pasaban and Niazmand (2021) observed that metformin limits intracellular ROS by activating the antioxidative system. Karthivashan et al. (2016) showed that Moringa powder and extract enriched with minerals, flavonoids, phenols, and minerals enhanced renal protection. Ahmed et al. (2020) found that moringa extract decreased serum creatinine and BUN in chronic kidney disease patients. Tang et al. (2017) also supported the role of ethanolic extract of *M. oleifera* in reducing low‐density lipoproteins (LDL) to inhibit oxidative stress and atherosclerosis in kidney infection. Ling and Kuo (2018) claimed that ethanolic extracts elevated creatinine clearance and limited plasma creatinine.

## 4 CONCLUSION

The present study shows that *M. oleifera* leaf powder and extract minimizes oxidative stress and reduces liver and kidney inflammation in female albino mice (*Mus musculus*). Furthermore, *M. oleifera* is nontoxic and enhances nephro‐hepatic activity in female albino mice with PCOS. The research demonstrated that administering *M. oleifera* at a dosage of 500 mg/kg BW in female albino mice was more effective in reducing the risk of liver and kidney diseases than metformin (250 mg/kg BW). The current study provided a direction that *M. oleifera* leaf powder and leaf extract have promising therapeutic potential for managing PCOS and infertility. However, the results of our research are limited to mice only, further studies with long‐term follow‐up and more diverse phenotypes are needed to endorse the potential of *M. oleifera* leaf powder and leaf extract to manage hepatic and renal damages caused by PCOS in human. Overall, this study highlights the potential of both *M. oleifera* leaf powder and extract as effective therapeutic options for managing PCOS, and liver and kidney diseases. It further promoted that *M. oleifera*‐based medication would be a better option to treat oxidative stress and several other risk factors linked with PCOS, and liver and kidney disorders.

## AUTHOR CONTRIBUTIONS

**Shakeela Khalid:** Resources (equal). **Muhammad Arshad:** Software (equal). **Shahid Mahmood:** Writing – original draft (equal). **Farzana Siddique:** Funding acquisition (equal). **Nida Aziz:** Methodology (equal). **Sarfraz Khan:** Visualization (equal). **Komal Raza:** Investigation (equal). **Waseem Khalid:** Funding acquisition (equal). **Ammar AL‐Farga:** Project administration (equal). **Faisal Aqlan:** Writing – review and editing (equal).

## FUNDING INFORMATION

This research did not receive any specific grant from funding agencies in the public, commercial, or non‐profit sectors.

## CONFLICT OF INTEREST STATEMENT

The authors have no conflict of interest to declare.

## ETHICS STATEMENT

All ethical principles were considered in this article.

## Supporting information


Tables S1–S11

## Data Availability

Samples of the compounds and data used during the current study are available from the corresponding author.

## References

[fsn33646-bib-0001] Abd‐Elhakim, Y. M. , Mohamed, W. A. , Bohi, K. M. E. , Ali, H. A. , Mahmoud, F. A. , & Saber, T. M. (2021). Prevention of melamine‐induced hepatorenal impairment by an ethanolic extract of *Moringa oleifera*: Changes in KIM‐1, TIMP‐1, oxidative stress, apoptosis, and inflammation‐related genes. Gene, 764, 145083. 32860902 10.1016/j.gene.2020.145083

[fsn33646-bib-0002] Adewusi, E. A. , & Afolayan, A. J. (2010). Effect of *Pelargonium reniforme* roots on alcohol‐induced liver damage and oxidative stress. Pharmaceutical Biology, 48(9), 980–987. 20731548 10.3109/13880200903410354

[fsn33646-bib-0003] Ahmadvand, H. , Tavafi, M. , Asadollahi, V. , Jafaripour, L. , Hadipour‐Moradi, F. , Mohammadrezaei‐Khoramabadi, R. , & Cheraghi, A. (2016). Protective effect of carvacrol on renal functional and histopathological changes in gentamicin‐induced‐nephrotoxicity in rats. Zahedan Journal of Research in Medical Sciences, 18(4), 1–5.

[fsn33646-bib-0004] Ahmed, N. F. , Sadek, K. M. , Soliman, M. K. , Khalil, R. H. , Khafaga, A. F. , Ajarem, J. S. , & Allam, A. A. (2020). *Moringa oleifera* leaf extract repairs the oxidative misbalance following sub‐chronic exposure to sodium fluoride in Nile tilapia *Oreochromis niloticus* . Animals, 10(4), 626. 32260525 10.3390/ani10040626 PMC7222772

[fsn33646-bib-0005] Ahmed, Z. , Ahmed, U. , Walayat, S. , Ren, J. , Martin, D. K. , Moole, H. , & Dhillon, S. (2018). Liver function tests in identifying patients with liver disease. Clinical and Experimental Gastroenterology, 11, 301–307. 30197529 10.2147/CEG.S160537 PMC6112813

[fsn33646-bib-0006] Akpan, H. B. , Akande, A. A. , Ojewale, A. O. , Oladipupo, F. E. , Akinpelu, O. F. , & Jimoh, S. O. (2018). *Moringa oleifera* ameliorates nephropathic changes in alloxaninduced diabetic adult Wistar rats. Journal of African Association of Physiological Sciences, 6(2), 110–118.

[fsn33646-bib-0007] Akter, T. , Rahman, M. A. , Moni, A. , Apu, M. , Islam, A. , Fariha, A. , & Uddin, M. J. (2021). Prospects for protective potential of *Moringa oleifera* against kidney diseases. Plants, 10(12), 2818. 34961289 10.3390/plants10122818 PMC8706354

[fsn33646-bib-0008] Allen, P. J. (2012). Creatine metabolism and psychiatric disorders: Does creatine supplementation have therapeutic value? Neuroscience & Biobehavioral Reviews, 36(5), 1442–1462. 22465051 10.1016/j.neubiorev.2012.03.005 PMC3340488

[fsn33646-bib-0009] Altaee, R. A. , & Fadheel, Q. J. (2021). The nephroprotective effects of *moringa oleifera* extract against contrast induced nephrotoxicity. Journal of Pharmaceutical Research International, 33, 63–70.

[fsn33646-bib-0010] Amelia, D. , Santoso, B. , Purwanto, B. , Miftahussurur, M. , & Joewono, H. T. (2018). Effects of *Moringa oleifera* on insulin levels and folliculogenesis in polycystic ovary syndrome model with insulin resistance. Immunology, Endocrine & Metabolic Agents in Medicinal Chemistry, 18(1), 22–30. 10.2174/1871522218666180426100754 PMC6174639 30369967

[fsn33646-bib-0011] Anesetti, G. , & Chávez‐Genaro, R. (2016). Neonatal testosterone exposure induces early development of follicular cysts followed by sympathetic ovarian hyperinnervation. Reproduction, Fertility and Development, 28(11), 1753–1761. 10.1071/RD14460 25989716

[fsn33646-bib-0012] Arafat, N. , Awadin, W. F. , El‐Shafei, R. A. , Farag, V. M. , & Saleh, R. M. (2018). Protective role of *Moringa oleifera* leaves extract against gentamicin‐induced nephro‐and hepato‐toxicity in chickens. The Alexandria Journal of Veterinary Sciences, 58, 173.

[fsn33646-bib-0013] Banafsheh, A. A. , & Sirous, G. (2016). Studies on oxidants and antioxidants with a brief glance at their relevance to the immune system. Life Sciences, 146, 163–173. 10.1016/j.lfs.2016.01.014 26792059

[fsn33646-bib-0014] Benjamini, Y. , & Braun, H. (2002). John W. Tukey's contributions to multiple comparisons. Annals of Statistics, 30, 1576–1594.

[fsn33646-bib-0015] Braunwald, E. , Fauci, A. S. , Hauser, S. L. , Longo, D. L. , & Jameson, J. L. (2005). Harrison's principles of internal medicine. McGraw‐Hill Companies, Inc.

[fsn33646-bib-0016] Broskey, N. T. , Tam, C. S. , Sutton, E. F. , Altazan, A. D. , Burton, J. H. , Ravussin, E. , & Redman, L. M. (2018). Metabolic inflexibility in women with PCOS is similar to women with type 2 diabetes. Nutrition & Metabolism, 15, 1–9. 30377436 10.1186/s12986-018-0312-9 PMC6195988

[fsn33646-bib-0017] Buege, J. A. , & Aust, S. D. (1978). Microsomal lipid peroxidation. In Methods in enzymology (Vol. 52, pp. 302–310). Academic press. 672633 10.1016/s0076-6879(78)52032-6

[fsn33646-bib-0018] Cadet, J. , Ravanat, J. L. , Tavernaporro, M. , Menoni, H. , & Angelov, D. (2012). Oxidatively generated complex DNA damage: Tandem and clustered lesions. Cancer Letters, 327, 5–15. 10.1016/j.canlet.2012.04.005 22542631

[fsn33646-bib-0019] Castro, A. F. , & Coresh, J. (2009). CKD surveillance using laboratory data from the population‐based National Health and nutrition examination survey (NHANES). American Journal of Kidney Diseases, 53(3), S46–S55. 19231761 10.1053/j.ajkd.2008.07.054 PMC2677815

[fsn33646-bib-0020] Chen, C. (2016). Sinapic acid and its derivatives as medicine in oxidative stress‐induced diseases and aging. Oxidative Medicine and Cellular Longevity, 12, 668708. 10.1155/2016/3571614 PMC4812465 27069529

[fsn33646-bib-0021] Chukwunonso Obi, B. , Chinwuba Okoye, T. , Okpashi, V. E. , Nonye Igwe, C. , & Olisah Alumanah, E. (2016). Comparative study of the antioxidant effects of metformin, glibenclamide, and repaglinide in alloxan‐induced diabetic rats. Journal of Diabetes Research, 2016, 1635361. 26824037 10.1155/2016/1635361 PMC4707348

[fsn33646-bib-0022] Del Rio, D. , Stewart, A. J. , & Pellegrini, N. (2005). A review of recent studies on malondialdehyde as toxic molecule and biological marker of oxidative stress. Nutrition, Metabolism and Cardiovascular Diseases, 15(4), 316–328. 10.1016/j.numecd.2005.05.003 16054557

[fsn33646-bib-0023] Domecq, J. P. , Prutsky, G. , Mullan, R. J. , Sundaresh, V. , Wang, A. T. , Erwin, P. J. , Welt, C. , Ehrmann, D. , Montori, V. M. , & Murad, M. H. (2013). Adverse effects of the common treatments for polycystic ovary syndrome: A systematic review and meta‐analysis. The Journal of Clinical Endocrinology & Metabolism, 98(12), 4646–4654. 24092830 10.1210/jc.2013-2374 PMC5399491

[fsn33646-bib-0024] Don, B. R. , & Kaysen, G. (2004). Poor nutritional status and inflammation: Serum albumin: Relationship to inflammation and nutrition. In Seminars in dialysis (Vol. 17, pp. 432–437). Blackwell Science Inc. 15660573 10.1111/j.0894-0959.2004.17603.x

[fsn33646-bib-0025] Erbey, J. R. , Silberman, C. , & Lydick, E. (2000). Prevalence of abnormal serum alanine aminotransferase levels in obese patients and patients with type 2 diabetes. The American Journal of Medicine, 109(7), 588–590. 11063962 10.1016/s0002-9343(00)00602-1

[fsn33646-bib-0026] Escobar‐Morreale, H. F. (2018). Polycystic ovary syndrome: Definition, aetiology, diagnosis and treatment. Nature Reviews Endocrinology, 14(5), 270–284. 10.1038/nrendo.2018.24 29569621

[fsn33646-bib-0027] Esmaeilzadeh, M. , Heidarian, E. , Shaghaghi, M. , Roshanmehr, H. , Najafi, M. , Moradi, A. , & Nouri, A. (2020). Gallic acid mitigates diclofenac‐induced liver toxicity by modulating oxidative stress and suppressing IL‐1β gene expression in male rats. Pharmaceutical Biology, 58(1), 590–596. 32633182 10.1080/13880209.2020.1777169 PMC7470116

[fsn33646-bib-0028] Fraser, S. D. , Roderick, P. J. , May, C. R. , McIntyre, N. , McIntyre, C. , Fluck, R. J. , & Taal, M. W. (2015). The burden of comorbidity in people with chronic kidney disease stage 3: A cohort study. BMC Nephrology, 16(1), 1–11. 10.1186/s12882-015-0189-z PMC4666158 26620131

[fsn33646-bib-0029] Gopalakrishnan, L. , Doriya, K. , & Kumar, D. S. (2016). *Moringa oleifera*: A review on nutritive importance and its medicinal application. Food Science and Human Wellness, 5(2), 49–56.

[fsn33646-bib-0030] Grosshagauer, S. , Pirkwieser, P. , Kraemer, K. , & Somoza, V. (2021). The future of moringa foods: A food chemistry perspective. Frontiers in Nutrition, 8, 751076. 34796194 10.3389/fnut.2021.751076 PMC8594418

[fsn33646-bib-0031] Hill, L. A. , Bodnar, T. S. , Weinberg, J. , & Hammond, G. L. (2016). Corticosteroid‐binding globulin is a biomarker of inflammation onset and severity in female rats. The Journal of Endocrinology, 230(2), 215–225. 27418032 10.1530/JOE-16-0047 PMC5338597

[fsn33646-bib-0032] Huang, Y. , Sun, J. , Wang, X. , Tao, X. , Wang, H. , & Tan, W. (2015). Asymptomatic chronic gastritis decreases metformin tolerance in patients with type 2 diabetes. Journal of Clinical Pharmacy and Therapeutics, 40(4), 461–465. 26032654 10.1111/jcpt.12290

[fsn33646-bib-0033] Iweala, E. E. , & Okeke, C. U. (2005). Comparative study of the hypoglycemic and biochemical effects of *Catharanthus roseus* (Linn) G. Apocynaceae (*Madagascar periwinkle*) and chlorpropamide (diabenese) on alloxan‐induced diabetic rats. Biokemistri, 17, 149–156.

[fsn33646-bib-0034] Jaccard, J. , Becker, M. A. , & Wood, G. (1984). Pairwise multiple comparison procedures: A review. Psychological Bulletin, 96(3), 589–596. 10.1037/0033-2909.96.3.589

[fsn33646-bib-0035] Javed, Z. , Papageorgiou, M. , Deshmukh, H. , Kilpatrick, E. S. , Mann, V. , Corless, L. , & Sathyapalan, T. (2019). A randomized, controlled trial of vitamin D supplementation on cardiovascular risk factors, hormones, and liver markers in women with polycystic ovary syndrome. Nutrients, 11(1), 188. 30658483 10.3390/nu11010188 PMC6356309

[fsn33646-bib-0036] Jelodar, G. , & Askari, K. (2012). Effect of Vitex agnus‐castus fruits hydroalcoholic extract on sex hormones in rat with induced polycystic ovary syndrome (PCOS). Physiology and Pharmacology, 16(1), 62–69.

[fsn33646-bib-0102] Jendrasskik, J. , & Grof, P. (1938). In vitro determination of total and direct bilirubin in serum or plasma. Biochemistry, 297(1), 81–89.

[fsn33646-bib-0037] Kagbo, H. D. , & Abaekwume, C. O. (2021). Hepato‐renal‐curative effect of the herbal supplement of Aloe vera Linn gel versus *Moringa oleifera* on acetaminophen‐induced damage on the liver and kidney of Wistar rats (*Rattus norvegicus*). Journal of Advances in Medical and Pharmaceutical Sciences, 23(1), 12–23.

[fsn33646-bib-0038] Kakalij, R. M. , Alla, C. P. , Kshirsagar, R. P. , Kumar, B. H. , Mutha, S. S. , & Diwan, P. V. (2014). Ameliorative effect of *Elaeocarpus ganitrus* on gentamicin‐induced nephrotoxicity in rats. Indian Journal of Pharmacology, 46(3), 298–302. 24987177 10.4103/0253-7613.132163 PMC4071707

[fsn33646-bib-0039] Kalhori, Z. , Mehranjani, M. S. , Azadbakht, M. , & Shariaatzadeh, M. A. (2018). Ovary stereological features and serum biochemical factors following induction of polycystic ovary syndrome with testosterone enanthate in mice: An experimental study. International Journal of Reproductive Bio Medicine, 16(4), 267–274. PMC6004592 29942935

[fsn33646-bib-0040] Karmanova, E. E. , Chernikov, A. V. , Popova, N. R. , Sharapov, M. G. , Ivanov, V. E. , & Bruskov, V. I. (2023). Metformin mitigates radiation toxicity exerting antioxidant and genoprotective properties. Naunyn‐Schmiedeberg's Archives of Pharmacology, 395, 1–12. 10.1007/s00210-023-02466-w PMC10036983 36961549

[fsn33646-bib-0041] Karthivashan, G. , Kura, A. U. , Arulselvan, P. , Isa, N. M. , & Fakurazi, S. (2016). The modulatory effect of *Moringa oleifera* leaf extract on endogenous antioxidant systems and inflammatory markers in an acetaminophen‐induced nephrotoxic mice model. PeerJ, 4, e2127. 27441110 10.7717/peerj.2127 PMC4941779

[fsn33646-bib-0042] Khalid, S. , Arshad, M. , Mahmood, S. , Siddique, F. , Roobab, U. , Ranjha, M. M. A. N. , & Lorenzo, J. M. (2023). Extraction and quantification of *Moringa oleifera* leaf powder extracts by HPLC and FTIR. Food Analytical Methods, 16, 1–11.

[fsn33646-bib-0043] Khalofah, A. , Bokhari, N. A. , Migdadi, H. M. , & Alwahibi, M. S. (2020). Antioxidant responses and the role of *Moringa oleifera* leaf extract for mitigation of cadmium stressed Lepidium sativum L. South African Journal of Botany, 129, 341–346.

[fsn33646-bib-0044] Kostoff, R. N. , Heroux, P. , Aschner, M. , & Tsatsakis, A. (2020). Adverse health effects of 5G mobile networking technology under real‐life conditions. Toxicology Letters, 323, 35–40. 31991167 10.1016/j.toxlet.2020.01.020

[fsn33646-bib-0045] Kumar, S. , & Pandey, A. K. (2013). Chemistry and biological activities of flavonoids: An overview. Scientific World Journal, 2013, 16. 10.1155/2013/162750 PMC3891543 24470791

[fsn33646-bib-0046] Lee, M. (Ed.). (2009). Basic skills in interpreting laboratory data. ASHP.

[fsn33646-bib-0047] Ling, X. C. , & Kuo, K. L. (2018). Oxidative stress in chronic kidney disease. Renal Replacement Therapy, 4(1), 1–9.

[fsn33646-bib-0101] Lowry, O. H. , Rosebrough, N. J. , Farr, A. L. , & Randall, R. J. (1951). Protein measurement with the Folin‐Phenol reagents. Journal of Biological Chemistry, 193(1), 265–275. 14907713

[fsn33646-bib-0048] MacFarlane, I. , Bomford, A. , & Sherwood, R. (2000). Liver disease and laboratory medicine. ACB Venture Publications.

[fsn33646-bib-0049] Manikandan, R. , Beulaja, M. , Thiagarajan, R. , Priyadarsini, A. , Saravanan, R. , & Arumugam, M. (2011). Ameliorative effects of curcumin against renal injuries mediated by inducible nitric oxide synthase and nuclear factor kappa B during gentamicin‐induced toxicity in Wistar rats. European Journal of Pharmacology, 670(2–3), 578–585. 21925163 10.1016/j.ejphar.2011.08.037

[fsn33646-bib-0050] McClatchey, K. D. (Ed.). (2002). Clinical laboratory medicine. Lippincott Williams & Wilkins.

[fsn33646-bib-0051] Meyer, E. J. , Nenke, M. A. , Rankin, W. , Lewis, J. G. , & Torpy, D. J. (2016). Corticosteroid‐binding globulin: A review of basic and clinical advances. Hormone and Metabolic Research, 48(6), 359–371. 27214312 10.1055/s-0042-108071

[fsn33646-bib-0052] Murri, M. , Luque‐Ramírez, M. , Insenser, M. , Ojeda‐Ojeda, M. , & Escobar‐Morreale, H. F. (2013). Circulating markers of oxidative stress and polycystic ovary syndrome (PCOS): A systematic review and meta‐analysis. Human Reproduction Update, 19(3), 268–288. 23303572 10.1093/humupd/dms059

[fsn33646-bib-0053] Nwankwo, N. E. , Nwodo, O. F. C. , Amalunweze, A. E. , Agbo, K. U. , & Abugu, S. C. (2015). Liver and kidney function tests and histological study on malaria parasite infected mice administered with seed extract of *Picralima nitida* . International Journal of Biochemistry Research & Review, 8(2), 1–14.

[fsn33646-bib-0054] Ojan, H. , & Nihorimbere, V. (2004). Antioxidant power of phytochemicals from *Psidium guajava* . Journal of Zhejiang University of Science, 5, 676–683. 10.1007/BF02840979 15101101

[fsn33646-bib-0055] Padureanu, R. , Albu, C. V. , Mititelu, R. R. , Bacanoiu, M. V. , Docea, A. O. , Calina, D. , & Buga, A. M. (2019). Oxidative stress and inflammation interdependence in multiple sclerosis. Journal of Clinical Medicine, 8(11), 1815. 31683787 10.3390/jcm8111815 PMC6912446

[fsn33646-bib-0056] Pasaban, M. , & Niazmand, S. (2021). The comparison of antioxidant effect of aspirin, metformin, atorvastatin and captopril Co‐administration in the heart and kidney tissues of diabetic rats. Iranian Journal of Pharmaceutical Research, 20(1), 27. 34400938 10.22037/ijpr.2019.112004.13481 PMC8170761

[fsn33646-bib-0057] Patil, C. N. , Racusen, L. C. , & Reckelhoff, J. F. (2017). Consequences of advanced aging on renal function in chronic hyperandrogenemic female rat model: Implications for aging women with polycystic ovary syndrome. Physiological Reports, 5(20), e13461. 29051304 10.14814/phy2.13461 PMC5661229

[fsn33646-bib-0058] Pizzino, G. , Irrera, N. , Cucinotta, M. , Pallio, G. , Mannino, F. , Arcoraci, V. , Squadrito, F. , Altavilla, D. , & Bitto, A. (2017). Oxidative stress: Harms and benefits for human health. Oxidative Medicine and Cellular Longevity, 2017, 1–13. 10.1155/2017/8416763 PMC5551541 28819546

[fsn33646-bib-0100] Reitman, S. , & Frankel, S. (1957). A colorimetric method for the determination of serum glutamic oxalacetic and glutamic pyruvic transaminases. American Journal of Clinical Pathology, 28(1), 56–63. 13458125 10.1093/ajcp/28.1.56

[fsn33646-bib-0059] Rojas, J. , Chávez, M. , Olivar, L. , Rojas, M. , Morillo, J. , Mejías, J. , & Bermúdez, V. (2014). Polycystic ovary syndrome, insulin resistance, and obesity: Navigating the pathophysiologic labyrinth. International Journal of Reproductive Medicine, 2014, 719050. 25763405 10.1155/2014/719050 PMC4334071

[fsn33646-bib-0061] Ruhl, C. E. , & Everhart, J. E. (2003). Determinants of the association of overweight with elevated serum alanine aminotransferase activity in the United States. Gastroenterology, 124(1), 71–79. 12512031 10.1053/gast.2003.50004

[fsn33646-bib-0062] Saleh, A. S. (2019). Evaluation of hepatorenal protective activity of *Moringa oleifera* on histological and biochemical parameters in cadmium intoxicated rats. Toxin Reviews, 38(4), 338–345.

[fsn33646-bib-0063] Saravanan, R. , Viswanathan, P. , & Pugalendi, K. V. (2006). Protective effect of ursolic acid on ethanol‐mediated experimental liver damage in rats. Life Sciences, 78, 713–718. 16137716 10.1016/j.lfs.2005.05.060

[fsn33646-bib-0064] Sawyer, S. F. (2009). Analysis of variance: The fundamental concepts. Journal of Manual & Manipulative Therapy, 17(2), 27E–38E.

[fsn33646-bib-0065] Schiødt, F. V. (2008). Gc‐globulin in liver disease. Danish Medical Bulletin, 55(3), 131–146. 19232164

[fsn33646-bib-0066] Schwimmer, J. B. , Khorram, O. , Chiu, V. , & Schwimmer, W. B. (2005). Abnormal aminotransferase activity in women with polycystic ovary syndrome. Fertility and Sterility, 83(2), 494–497. 15705403 10.1016/j.fertnstert.2004.08.020

[fsn33646-bib-0067] Shah, K. N. , & Patel, S. S. (2016). Phosphatidylinositide 3‐kinase inhibition: A new potential target for the treatment of polycystic ovarian syndrome. Pharmaceutical Biology, 54(6), 975–983. 26459667 10.3109/13880209.2015.1091482 PMC11133948

[fsn33646-bib-0068] Siahaan, S. C. P. , Santoso, B. , & Widjiati, S. (2022). Effectiveness of *Moringa oleifera* leaves on TNF‐α expression, insulin levels, glucose levels and follicle count in *Rattus norvegicus* PCOS model. Diabetes, Metabolic Syndrome and Obesity: Targets and Therapy, 29, 3255–3270. 10.2147/DMSO.S385492 PMC9595062 36304481

[fsn33646-bib-0069] Singh, B. N. , Singh, B. R. , Singh, R. L. , Prakash, D. , Dhakarey, R. , Upadhyay, G. , & Singh, H. B. (2009). Oxidative DNA damage protective activity, antioxidant and anti‐quorum sensing potentials of *Moringa oleifera* . Food and Chemical Toxicology, 47(6), 1109–1116. 19425184 10.1016/j.fct.2009.01.034

[fsn33646-bib-0070] Singha, P. K. , Roy, S. , & Dey, S. (2007). Protective activity of andrographolide and arabinogalactan proteins from *Andrographis paniculata* Nees. Against ethanol‐induced toxicity in mice. Journal of Ethnopharmacology, 111(1), 13–21. 17127022 10.1016/j.jep.2006.10.026

[fsn33646-bib-0071] Sirmans, S. M. , & Pate, K. A. (2014). Epidemiology, diagnosis, and management of polycystic ovary syndrome. Clinical Epidemiology, 6, 1–13. 10.2147/CLEP.S37559 PMC3872139 24379699

[fsn33646-bib-0072] Song, Y. , Ye, W. , Ye, H. , Xie, T. , Shen, W. , & Zhou, L. (2019). Serum testosterone acts as a prognostic indicator in polycystic ovary syndrome‐associated kidney injury. Physiological Reports, 7(16), e14219. 31448581 10.14814/phy2.14219 PMC6709419

[fsn33646-bib-0073] Tang, Y. , Choi, E. J. , Han, W. C. , Oh, M. , Kim, J. , Hwang, J. Y. , & Kim, E. K. (2017). *Moringa oleifera* from Cambodia ameliorates oxidative stress, hyperglycemia, and kidney dysfunction in type 2 diabetic mice. Journal of Medicinal Food, 20(5), 502–510. 28467233 10.1089/jmf.2016.3792

[fsn33646-bib-0074] Targher, G. , Rossini, M. , & Lonardo, A. (2016). Evidence that non‐alcoholic fatty liver disease and polycystic ovary syndrome are associated by necessity rather than chance: A novel hepato‐ovarian axis? Endocrine, 51, 211–221. 26024975 10.1007/s12020-015-0640-8

[fsn33646-bib-0075] Tee‐ngam, P. , Nunant, N. , Rattanarat, P. , Siangproh, W. , & Chailapakul, O. (2013). Simple and rapid determination of ferulic acid levels in food and cosmetic samples using paper‐based platforms. Sensors, 13(10), 13039–13053. 24077320 10.3390/s131013039 PMC3859048

[fsn33646-bib-0076] Thapa, B. R. , & Walia, A. (2007). Liver function tests and their interpretation. The Indian Journal of Pediatrics, 74, 663–671. 17699976 10.1007/s12098-007-0118-7

[fsn33646-bib-0103] Tietz, N. W. (1994). Textbook of clinical chemistry (2nd ed.). W.B. Saunders Company.

[fsn33646-bib-0077] Traynor, J. , Mactier, R. , Geddes, C. C. , & Fox, J. G. (2006). How to measure renal function in clinical practice. BMJ, 333(7571), 733–737. 17023465 10.1136/bmj.38975.390370.7C PMC1592388

[fsn33646-bib-0078] Tsatsakis, A. , Docea, A. O. , Calina, D. , Tsarouhas, K. , Zamfira, L. M. , Mitrut, R. , & Neagu, M. (2019). A mechanistic and pathophysiological approach for stroke associated with drugs of abuse. Journal of Clinical Medicine, 8(9), 1295. 31450861 10.3390/jcm8091295 PMC6780697

[fsn33646-bib-0079] Vassilatou, E. (2014). Nonalcoholic fatty liver disease and polycystic ovary syndrome. World Journal of Gastroenterology, 20(26), 8351–8363. 25024594 10.3748/wjg.v20.i26.8351 PMC4093689

[fsn33646-bib-0080] Velaga, V. , Suryadevara, N. , Chee, L. L. , & Ismail, N. E. (2017). Phytochemical analysis and immuno‐modulatory effect of *Moringa oleifera* flowers. Journal of Pharmacy & Pharmaceutical Sciences, 9, 24–28.

[fsn33646-bib-0081] Vergara‐Jimenez, M. , Almatrafi, M. M. , & Fernandez, M. L. (2017). Bioactive components in *Moringa oleifera* leaves protect against chronic disease. Antioxidants, 6(4), 91. 29144438 10.3390/antiox6040091 PMC5745501

[fsn33646-bib-0082] Verzola, D. , Gandolfo, M. T. , Salvatore, F. , Villaggio, B. , Gianiorio, F. , Traverso, P. , & Garibotto, G. (2004). Testosterone promotes apoptotic damage in human renal tubular cells. Kidney International, 65(4), 1252–1261. 15086464 10.1111/j.1523-1755.2004.00497.x

[fsn33646-bib-0083] Webster, A. C. , Nagler, E. V. , Morton, R. L. , & Masson, P. (2017). Chronic kidney disease. The Lancet, 389(10075), 1238–1252. 10.1016/S0140-6736(16)32064-5 27887750

[fsn33646-bib-0084] Wong, H. Y. , Tan, J. Y. L. , & Lim, C. C. (2004). Abnormal liver function tests in the symptomatic pregnant patient: The local experience in Singapore. Annals‐Academy of Medicine Singapore, 33(2), 204–208. 15098635

[fsn33646-bib-0085] Yang, D. , Shen, J. , Huang, H. , Wang, J. , Sun, F. , Zeng, T. , & Weng, Y. (2022). Elevated albumin to globulin ratio on day 7 is associated with improved function outcomes in acute ischemic stroke patients with intravenous thrombolysis. Journal of Inflammation Research, 15, 2695–2705. 35505797 10.2147/JIR.S347026 PMC9057231

[fsn33646-bib-0086] Yoshino, Y. , Taguchi, A. , Shimizuguchi, T. , Nakajima, Y. , Takao, M. , Kashiyama, T. , & Yasugi, T. (2019). A low albumin to globulin ratio with a high serum globulin level is a prognostic marker for poor survival in cervical cancer patients treated with radiation based therapy. International Journal of Gynecologic Cancer, 29(1), 17–22. 10.1136/ijgc-2018-000025 30640678

[fsn33646-bib-0087] Younas, A. , Hussain, L. , Shabbir, A. , Asif, M. , Hussain, M. , & Manzoor, F. (2022). Effects of fagonia indica on letrozole‐induced polycystic ovarian syndrome (PCOS) in young adult female rats. Evidence‐Based Complementary and Alternative Medicine, 7, 1397060. 10.1155/2022/1397060 PMC9162856 35664938

[fsn33646-bib-0088] Young, D. S. (1997). Effect of preanalytical variables on laboratory tests (2nd ed.). AACC press.

[fsn33646-bib-0089] Zduńska, K. , Dana, A. , Kolodziejczak, A. , & Rotsztejn, H. (2018). Antioxidant properties of ferulic acid and its possible application. Skin Pharmacology and Physiology, 31(6), 332–336. 30235459 10.1159/000491755

[fsn33646-bib-0090] Zucca, P. , Argiolas, A. , Sanna, F. , Nieddu, M. , Sollai, F. A. , Pintus, M. , & Rescigno, A. (2016). Biological activities and nutraceutical potentials of water extracts from different parts of *Cynomorium coccineum* L.(Maltese mushroom). Polish Journal of Food and Nutrition Sciences, 66(3), 179–188.

