# Supplementary material for: Assessment of hepatoprotective, nephroprotective efficacy, and antioxidative potential of Moringa oleifera leaf powder and ethanolic extract against PCOS‐induced female albino mice (Mus Musculus)
Source: Food Sci Nutr. 2023 Sep 1;11(11):7206–17. doi: 10.1002/fsn3.3646 (PMC10630814; doi:10.1002/fsn3.3646)
Supplement: Supplementary file 1 — Tables S1–S11 [file FSN3-11-7206-s001.docx]

**Supplementary Material**

| Source of Variation | Df | SS | MS | F | *P* |
| --- | --- | --- | --- | --- | --- |
| Treatments (T) | 6 | 10864898 | 1810816 | 2428.61 | 0.00** |
| Days (D) | 2 | 2768413 | 1384207 | 1856.46 | 0.00** |
| T × D | 12 | 3902776 | 325231 | 436.19 | 0.00** |
| Error | 189 | 140922 | 746 |  |  |
| Total | 209 | 17677009 |  |  |  |

Table 1. Analysis of variance indicating the significance of treatments and days on serum MDA (nM/ml).

** Highly significant at *P* ≤ 0.01, *Significant at P ≤ 0.05, ns = nonsignificant

| Source of Variation | Df | SS | MS | F | *P* |
| --- | --- | --- | --- | --- | --- |
| Treatments (T) | 6 | 16211 | 2701.86 | 225.85 | 0.00** |
| Days (D) | 2 | 3341 | 1670.74 | 139.66 | 0.00** |
| T × D | 12 | 3355 | 279.59 | 23.37 | 0.00** |
| Error | 189 | 2261 | 11.96 |  |  |
| Total | 209 | 25169 |  |  |  |

Table 2. Analysis of variance indicating the significance of treatments and days on serum AST (U/l).

** Highly significant at *P* ≤ 0.01, *Significant at P ≤ 0.05, ^ns^ = nonsignificant

Table 3. Analysis of variance indicating the significance of treatments and days on serum ALT (U/l).

| Source of Variation | Df | SS | MS | F | *P* |
| --- | --- | --- | --- | --- | --- |
| Treatments (T) | 6 | 22721 | 3786.92 | 280.38 | 0.00** |
| Days (D) | 2 | 5083 | 2541.63 | 188.18 | 0.00** |
| T × D | 12 | 4245 | 353.73 | 26.19 | 0.00** |
| Error | 189 | 2553 | 13.51 |  |  |
| Total | 209 | 34602 |  |  |  |

** Highly significant at *P* ≤ 0.01, *Significant at P ≤ 0.05, ^ns^ = non-significant

Table 4. Analysis of variance indicating the significance of treatments and days on serum bilirubin total (mg/dl).

| Source of Variation | Df | SS | MS | F | *P* |
| --- | --- | --- | --- | --- | --- |
| Treatments (T) | 6 | 13.461 | 2.24 | 372.28 | 0.00** |
| Days (D) | 2 | 1.126 | 0.56 | 93.43 | 0.00** |
| T × D | 12 | 2.190 | 0.18 | 30.28 | 0.00** |
| Error | 189 | 1.139 | 0.00 |  |  |
| Total | 209 | 17.916 |  |  |  |

** Highly significant at *P* ≤ 0.01, *Significant at P ≤ 0.05, ^ns^ = non-significant

Table 5. Analysis of variance indicating the significance of treatments and days on serum ALP (U/l).

| Source of Variation | Df | SS | MS | F | *P* |
| --- | --- | --- | --- | --- | --- |
| Treatments (T) | 6 | 168729 | 28121.6 | 700.54 | 0.00** |
| Days (D) | 2 | 55320 | 27659.8 | 689.04 | 0.00** |
| T × D | 12 | 58204 | 4850.3 | 120.83 | 0.00** |
| Error | 189 | 7587 | 40.1 |  |  |
| Total | 209 | 289840 |  |  |  |

** Highly significant at *P* ≤ 0.01, *Significant at P ≤ 0.05, ^ns^ = nonsignificant

Table 6. Analysis of variance indicating the significance of treatments and days on serum total protein (g/dl).

| Source of Variation | Df | SS | MS | F | P |
| --- | --- | --- | --- | --- | --- |
| Treatments (T) | 6 | 147.20 | 24.53 | 448.54 | 0.00** |
| Days (D) | 2 | 1.20 | 0.60 | 11.04 | 0.00** |
| T × D | 12 | 67.89 | 5.65 | 103.44 | 0.00** |
| Error | 189 | 10.33 | 0.054 |  |  |
| Total | 209 | 226.65 |  |  |  |

** Highly significant at *P* ≤ 0.01, *Significant at P ≤ 0.05, ^ns^ = nonsignificant

Table 7. Analysis of variance indicating the significance of treatments and days on serum albumin (g/dl).

| Source of Variation | Df | SS | MS | F | P |
| --- | --- | --- | --- | --- | --- |
| Treatments (T) | 6 | 935.6 | 155.927 | 68.39 | 0.00** |
| Days (D) | 2 | 315.2 | 157.576 | 69.12 | 0.00** |
| T × D | 12 | 236.6 | 19.715 | 8.65 | 0.00** |
| Error | 189 | 430.9 | 2.280 |  |  |
| Total | 209 | 1918.20 |  |  |  |

** Highly significant at *P* ≤ 0.01, *Significant at P ≤ 0.05, ^ns^ = nonsignificant

Table 8. Analysis of variance indicating the significance of treatments and days on serum globulin (g/dl).

| Source of Variation | Df | SS | MS | F | P |
| --- | --- | --- | --- | --- | --- |
| Treatments (T) | 6 | 51.80 | 8.63 | 410.34 | 0.00** |
| Days (D) | 2 | 35.05 | 17.52 | 833.04 | 0.00** |
| T × D | 12 | 17.24 | 1.43 | 68.29 | 0.00** |
| Error | 189 | 3.97 | 0.02 |  |  |
| Total | 209 | 108.08 |  |  |  |

** Highly significant at *P* ≤ 0.01, *Significant at P ≤ 0.05, ^ns^ = non-significant

Table 9. Analysis of variance indicating the significance of treatments and days on serum A/G ratio.

| Source of Variation | Df | SS | MS | F | *P* |
| --- | --- | --- | --- | --- | --- |
| Treatments (T) | 6 | 9.56 | 1.59 | 196.37 | 0.00** |
| Days (D) | 2 | 1.27 | 0.63 | 78.21 | 0.00** |
| T × D | 12 | 3.73 | 0.31 | 38.32 | 0.00** |
| Error | 189 | 1.53 | 0.00 |  |  |
| Total | 209 | 16.10 |  |  |  |

** Highly significant at *P* ≤ 0.01, *Significant at P ≤ 0.05, ^ns^ = nonsignificant

Table 10. Analysis of variance indicating the significance of treatments and days on serum urea (mg/dl).

| Source of Variation | Df | SS | MS | F | *P* |
| --- | --- | --- | --- | --- | --- |
| Treatments (T) | 6 | 9942 | 1656.99 | 180.85 | 0.00** |
| Days (D) | 2 | 1428 | 714.10 | 77.94 | 0.00** |
| T × D | 12 | 5012 | 417.64 | 45.58 | 0.00** |
| Error | 189 | 1732 | 9.16 |  |  |
| Total | 209 | 18114 |  |  |  |

** Highly significant at *P* ≤ 0.01, *Significant at P ≤ 0.05, ^ns^ = nonsignificant

Table 11. Analysis of variance indicating the significance of treatments and days on serum creatinine (mg/dl).

| Source of Variation | Df | SS | MS | F | *P* |
| --- | --- | --- | --- | --- | --- |
| Treatments (T) | 6 | 14.19 | 2.36 | 293.30 | 0.00** |
| Days (D) | 2 | 0.71 | 0.35 | 44.30 | 0.00** |
| T × D | 12 | 1.29 | 0.10 | 13.33 | 0.00** |
| Error | 189 | 1.52 | 0.00 |  |  |
| Total | 209 | 17.73 |  |  |  |

** Highly significant at *P* ≤ 0.01, *Significant at P ≤ 0.05, ^ns^ = nonsignificant
